# Identification of Novel Factors Involved in Modulating Motility of *Salmonella enterica* Serotype Typhimurium

**DOI:** 10.1371/journal.pone.0111513

**Published:** 2014-11-04

**Authors:** Lydia M. Bogomolnaya, Lindsay Aldrich, Yuri Ragoza, Marissa Talamantes, Katharine D. Andrews, Michael McClelland, Helene L. Andrews-Polymenis

**Affiliations:** 1 Department of Microbial Pathogenesis and Immunology, College of Medicine, Texas A&M University, Bryan, Texas, United States of America; 2 Institute of Fundamental Medicine and Biology, Kazan Federal University, Kazan, Russia; 3 Department of Microbiology and Molecular Genetics, University of California Irvine, Irvine, California, United States of America; University of Osnabrueck, Germany

## Abstract

*Salmonella enterica* serotype Typhimurium can move through liquid using swimming motility, and across a surface by swarming motility. We generated a library of targeted deletion mutants in *Salmonella* Typhimurium strain ATCC14028, primarily in genes specific to *Salmonella*, that we have previously described. In the work presented here, we screened each individual mutant from this library for the ability to move away from the site of inoculation on swimming and swarming motility agar. Mutants in genes previously described as important for motility, such as *flgF, motA, cheY* are do not move away from the site of inoculation on plates in our screens, validating our approach. Mutants in 130 genes, not previously known to be involved in motility, had altered movement of at least one type, 9 mutants were severely impaired for both types of motility, while 33 mutants appeared defective on swimming motility plates but not swarming motility plates, and 49 mutants had reduced ability to move on swarming agar but not swimming agar. Finally, 39 mutants were determined to be hypermotile in at least one of the types of motility tested. Both mutants that appeared non-motile and hypermotile on plates were assayed for expression levels of FliC and FljB on the bacterial surface and many of them had altered levels of these proteins. The phenotypes we report are the first phenotypes ever assigned to 74 of these open reading frames, as they are annotated as ‘hypothetical genes’ in the Typhimurium genome.

## Introduction

Infection with *Salmonella enterica* serotypes remains a serious human and animal health problem worldwide. *Salmonella*e cause an array of diseases ranging from gastroenteric disease to systemic disease including Typhoid fever and bacteremia [1]. While gastroenteritis as a result of *Salmonella* infection is common worldwide, systemic diseases caused by this organism are relatively rare in the developed world. Serotype Typhimurium is one of the two most common serotypes identified from cases of clinical disease in the United States [2]. After gaining access to a susceptible host by the oro-fecal route, *Salmonella* utilizes multiple strategies to colonize and persist. *Salmonellae* have many well studied virulence factors including Type 3 Secretion Systems (T3SS-1 and -2), lipopolysaccharides (LPS), fimbria and others, and are capable of multiple types of motility including swimming and swarming. Both types of motility require the presence of functional flagella that are composed of many proteins and consist of the following structures: the basal body, the hook and the filament. Flagellar biosynthesis is a complex, tightly regulated process where gene products are produced in the order of flagellar apparatus assembly. The current understanding of flagella structure and regulation in *Salmonella* is nicely summarized in recent review by Chevance and Hughes [3]. Motility is linked to virulence in many pathogenic bacteria [4], [5].

Swimming motility is directed movement through liquid, which is assayed using semi-solid “swimming” media, containing a low concentration of agar. Under these conditions individual bacteria swim through medium-filled spaces between agar [6]. Swimming motility is closely linked to chemotaxis [7], the ability to orient bacteria along certain chemical gradients, and is thought to allow bacteria to detect and pursue nutrients or avoid unwanted repellents [7]. Ultimately, this kind of motility allows these organisms to avoid unfavorable environments for colonization and to reach and maintain preferred niches for colonization.

Swarming motility is a multi-cellular phenomenon involving the coordinated and rapid movement of a bacterial population across a semisolid surface [8]. Factors on which swarming is known to depend include bacterial cell density, media composition and surface moistness [9]–[12]. Swarming is not just another form of motility but rather a part of alternative growth state and is characterized by change in gene expression of nearly one third of genome in *Salmonella* Typhimurium [4].

Recent large-scale studies have been performed to identify genetic determinants required for each type of motility in *E. coli*, an organism closely related to *Salmonella*. Both transposon mutants in *E.coli* K-12 [13], and the Keio collection, a collection of targeted deletion mutants in each non-essential open reading frame in *E. coli* K-12 [14], were screened to identify mutants with reduced motility. These comprehensive studies demonstrated that numerous genes are involved in regulation of motility in bacteria.

We have generated a library of 1023 targeted single gene deletion mutants (SGD) in virulent *Salmonella enterica* serotype Typhimurium ATCC14028s [15]. This library contains mutants in nearly all *Salmonella*-specific open reading frames as well as an additional 100 genes shared between *Salmonella* and other *Enterobacteriaceae.* We screened this collection to systematically identify those mutants in our collection that had either reduced or enhanced ability to move away from the site of inoculation on swimming and/or swarming motility agar. We identified 160 mutants with altered motility in at least one condition. Mutants with previously known motility defects (flagellar, LPS biosynthesis, chemotaxis genes) were correctly identified in our screen. We identified mutants in nine additional genes that are unable to move away from the site of inoculation on both types of agar. Furthermore, we found that the ability to swim and swarm could be uncoupled in some mutants. We also identified a significant number of mutants that had enhanced motility of one or both types (‘hypermotility’). Finally, many of the motility phenotypes we identified belong to mutants with mutations in genes annotated as of unknown function (FUN, or ‘orphan’ genes), and are thus the first phenotypes of any kind described for these genes.

## Materials and Methods

### Bacterial Strains and Media

All *Salmonella enterica* serotype Typhimurium strains used in this study were derived from ATCC14028, including HA420, a spontaneous Nalidixic acid resistant isolate [16]. Targeted deletion mutants screened in this study have been described previously [15].

*Salmonella* strains used in this study were routinely grown on LB agar or broth, or on M9 minimal media. The growth of all mutant strains was tested on minimal M9 media [17], prior to use of these mutants in motility assays. Antibiotics were added in the following concentrations as appropriate: 50 mg/L Kanamycin sulfate, 50 mg/L of Nalidixic acid [15], [16]. Media for assaying swimming and swarming motility have been described previously [11]. Swimming was assayed on plates containing 0.3% Difco Bacto Agar (LB Miller base 25 g/L), while swarming motility was assayed on 0.6% Difco Bacto Agar (LB Miller base 25 g/L, and 0.5% glucose).

### Screening Individual Mutants for Swimming and Swarming Motility

Our collection of targeted deletion mutants was assayed in 96-well format for both swimming and swarming on large agar plates (15 cm diameter). Strains were inoculated into the appropriate agar with 96-pin replicator, incubated at 37°C, and closely monitored for the duration of the assay. Wild type ATCC14028s and ATCC14028r (smooth and rough LPS) were included as positive and negative controls on each motility plate. Several hours post-inoculation (3.5 hours for swimming, 5 hours for swarming), the swimming and swarming ability of each strain was evaluated by estimating the diameter of the spread of the bacteria and assigning a motility score. Individuals scoring motility were blinded to the identity of the mutants being scored, but were aware of the location of positive and negative controls inoculated on every plate. Motility was scored on a scale of 0 (completely non-motile) to 10 (extremely hypermotile) relative to wild type ATCC14028s, which was always assigned a motility score of 5. Our scoring system allowed us to identify mutants with a range of hypo- and hyper- motility. Large-scale screening assays were performed in triplicate and were repeated on at least three separate occasions with the entire collection of mutants. The mean of the motility scores for a given assay were determined for each mutant and these are shown in Table S1. Mutants used in our large scale screening were not transduced into a new genetic background, thus motility phenotypes associated with gene loss will be confirmed in future studies.

Mutants with the most severe phenotypes (less than 25% motility of wild type or hypermotile mutants) were further evaluated by measuring the diameter of the swimming or swarming colony as compared to positive and negative controls. This step was required for identification and removal from the future study a few false positive motility candidates that during primary screening were scored as non-motile or hypermotile due to uneven transfer of cells with 96-pin replicator. Overnight cultures of each mutant were grown and normalized by OD_600_. 3 µl was spotted on motility plates, incubated and scored as described above. The diameter of each swimming or swarming colony was measured, and compared to positive (WT ATCC14028s, smooth) and negative (ATCC14028r, rough mutant or *motA*) control isolates. Experiments were conducted in triplicate, and were repeated on three separate occasions. The identity of each mutant that displayed a statistically significant phenotype in our motility assays was verified by PCR using primers flanking the site of deletion if not previously verified. Statistical significance was determined using a Student's *t*-test and a *p*-value of <0.05.

### Evaluation of Flagellin Expression

Each mutant with reduced or enhanced motility was evaluated to determine the level of FljB and FliC produced in the bacterial cell, and to determine the amount of each of these proteins that reach the bacterial surface as compared to the isogenic wild type organism. Bacterial cultures grown to stationary phase were normalized by OD_600_ and bacteria were collected by centrifugation (Eppendorf 5415D). In order to shear flagella from the bacterial surface, pelleted bacteria were resuspended in 1 ml PBS and subjected to 5 minutes of vortexing (Vortex-genie, Scientific Industries) [18]. Sheared protein was precipitated using Trichloroacetic acid (TCA) (6% final concentration) overnight at 4°C, washed twice with 300 µl of acetone and resuspended in SDS sample buffer. Precipitated protein from sheared fractions was evaluated by SDS-PAGE and Western Analysis using antibodies against FliC and FljB (Difco). The remaining bacterial pellet (without sheared flagella) was solubilized in SDS sample buffer and examined by 12% SDS-PAGE and Coomassie staining to ensure equivalent loading. Wild type organisms and *ΔfljB* and *ΔfliC* mutants were used as positive and negative controls in these assays.

## Results and Discussion

### Screening of a collection of targeted deletion mutants in *Salmonella* to identify determinants of motility

**Figure 1:**
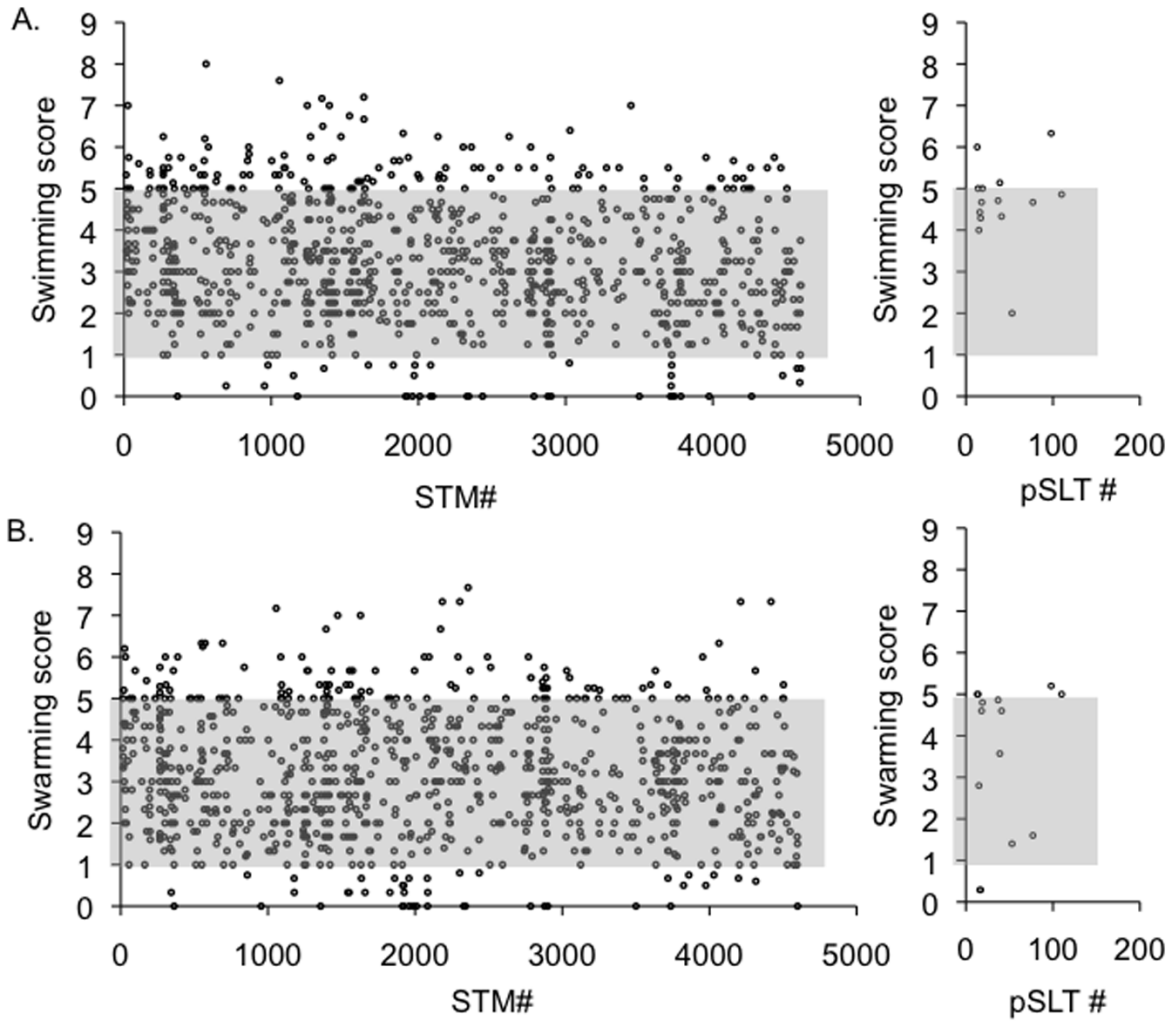
Motility scores of single deletion mutants of *Salmonella* Typhimurium ATCC14028 on swimming (A) and swarming (B) agar. Stationary phase cultures were transferred to motility agar, and motility was scored after 3.5 and 5 hours of incubation at 37°C on swimming or swarming plates, respectively. Motility was scored on the scale from 0 to 10 with wild type motility equal to 5. Data are presented as average swimming or swarming score from experiments with triplicate samples, performed on three independent occasions. Dots located outside of shaded area indicate scores greater than 5.25 and lower than 1.25 to define mutants with increased or reduced (<25 % of wild type) motility.

**Figure 2:**
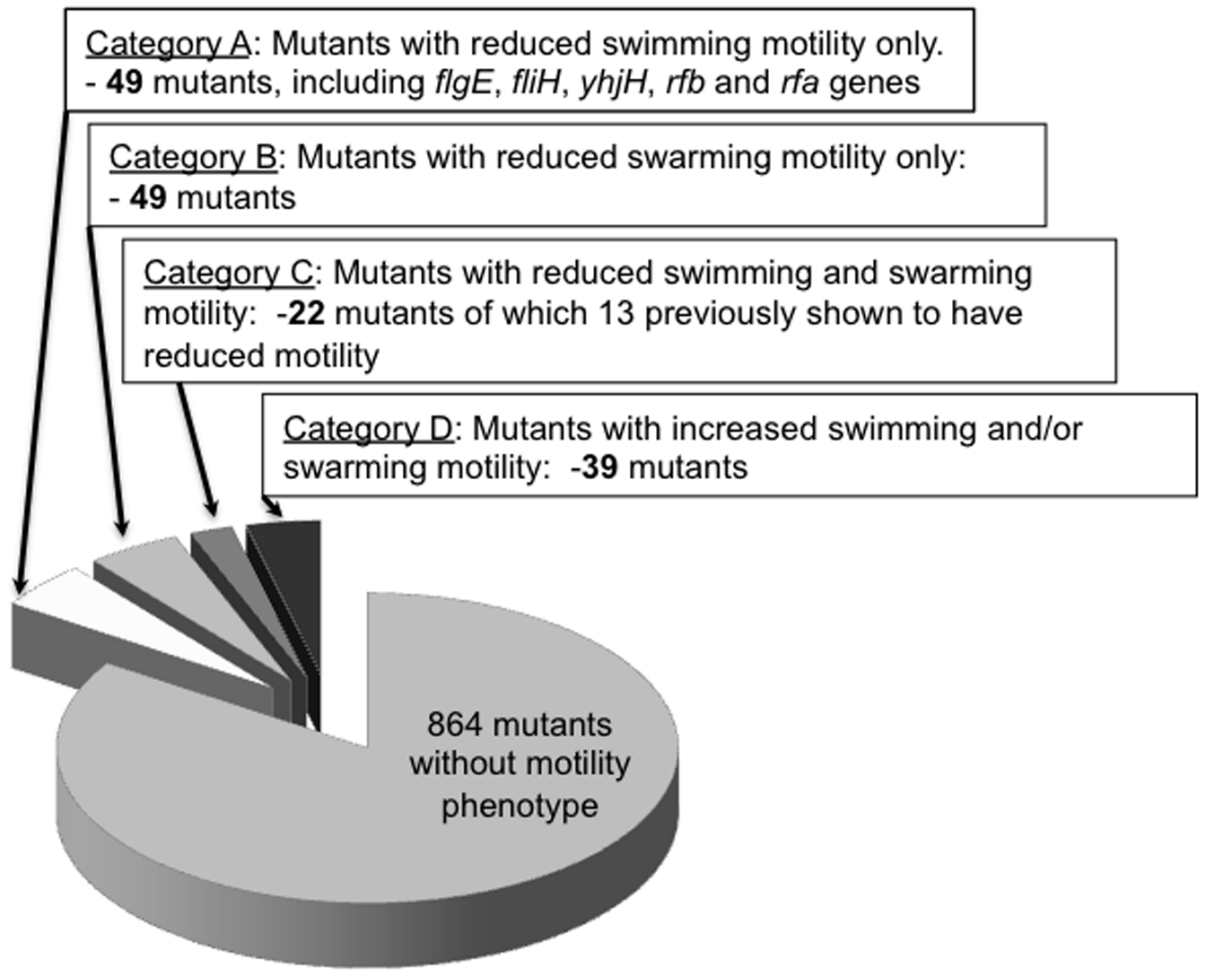
Distribution of motility phenotypes in single gene knock out collection.

We have screened a library of targeted single-gene deletion mutants in many genes that are specific to *Salmonellae* and not shared with close relatives, in addition to approximately 100 mutants in shared genes that served as controls, for motility phenotypes on plates [15] (Figure 1). We identified 160 mutants with altered motility. Of these mutants, only 29 were previously connected to motility either in *Salmonella* or *E. coli*. We divided the resulting set of mutants with motility phenotypes into four categories: (a) Mutants with defects in both types of motility; (b) Mutants with reduced swimming motility, swarming motility is unaffected; (c) Mutants reduced swarming motility only, swimming is unaffected; (d) Mutants that were hypermotile (Figure 2).

### Mutants with reduced swimming and swarming motility

We identified 21 mutants that moved less than or equal to 25% of wild type from the initial site of inoculation (i.e. impaired swimming and swarming). This group contains mutations in 12 genes previously reported to be needed for swimming and swarming motility, including flagellar genes *flhA, flhB*, *flgF*, *flgG*, *motA*, *motB*, LPS biosynthesis genes *rfbN*, *rfbK*, *rfaI*, chemotaxis gene *cheY* and others (Table 1), validating our approach. Our targeted deletion collection assayed in this study contained only eight mutants in known flagellar genes, and each of the mutants in these genes had the expected non-motile phenotype.

**Table 1:** Pathway clustering of mutants with altered motility.

Functional categories*	Swimming and swarming defect	Reduced swimming only	Reduced swarming only
Motility	***flgF*** ** **, ** ***flgG, flhA, flhB, motA, motB, fliD, fliM***	***flgE*** *, ssaV, * ***fliH*** *, stjC* ***	*ssaU, pefD, pefC*
Cell envelope biogenesis	***rfbN*** *, * ***rfaI***	***rfbP, rfbM, rfbJ, rfbC, rfbD, rfaJ, rfaG, rfaQ***	
Signal transduction	*STM0343, STM0551, * ***cheY*** *,*	*phoQ, * ***yhjH*** *, yjcC*	*yciR*
Carbohydrate transport & metabolism	***rfbK***	*STM0722, STM4424*	*STM0860, STM3780*
Transport and metabolism	*aroD*	*aroA, fur, yfeJ, pdxK, * ***tatC***	*yliB, STM1635, sfbA, mgtB, sodA, STM0163, STM1546, STM0857*
Transcription	*tctD*	*invF, STM2912, STM3696, STM4417, * ***arcA***	*STM0859, ydiP, torR, STM4315*
Replication, recombination and repair		*STM1005*	*STM1861*
Translation, ribosomal structure and biogenesis		*valS*	*STM1552*
Posttranslational modification, protein turnover, chaperones		*STM2743, sspA*	
Energy production and conversion			*STM0762, STM0858*
Cell cycle control, cell division, chromosome partitioning			*STM2594*
Defense mechanism			*STM4262*
Not in COGs	*STM0699, STM2010, STM2880, sthE, STM4595*	*STM0289, STM0295, STM0660, STM0971, STM1040, STM1331, ssaG, STM2374, sptP, sipA, STM3026, * ***rfaL*** *, yibR, * ***rfaP*** *, STM3783, STM4216,STM4219, sRNA candidate C1023*	*STM0056, STM0362, STM1131, pagC, STM1254, STM1258, STM1543, srfC, STM1632, STM1856, STM1926, STM1958, STM2303, STM2508, ygaU, STM3125, lpfE, STM3944, STM4030.S, STM4197, STM4204, STM4529, STM4574, STM4599, invR*

*Based on COGs (Clusters of Orthologous Groups of protein).

**Mutants with previously known motility phenotype are shown in bold.

***Mutants in fimbrial genes are underlined.

In addition, we identified general motility phenotypes for mutants in 9 genes not previously described to be involved in motility (Table 1). Four of these mutants had deletions in genes with putative functions in transport (*aroD*), in transcription (*tctD*) and in signal transduction (*STM0343*, *STM0551*). The remaining five mutants with reduced movement from the site of inoculation on swimming and swarming agar had deletion mutations in fimbrial genes *sthE*, *STM4595* [19], *STM0699*, *STM2010* and *STM2880*, a gene with unknown function encoded within SPI-1 [4], [20]. None of the mutants in this group had a growth defect in LB-broth (Figure S1). We note that our screening assay does not allow us to determine why mutants do not appear to spread from the site of inoculation. As such, classification of these mutants into categories such as defects in flagellar motility, motor activity, or chemotaxis is a fascinating area of future investigation.

We hypothesized that some of the phenotypes we observed could be due to an inability to export flagellin to the bacterial surface. The external portion of the flagellum is a helical filament composed of flagellin proteins, FliC or FljB [21]. In our wild type (ATCC14028) population FliC appeared to be the major flagellin expressed (accounts for>70 % of total flagellin) and FljB expression was weak (data not shown). We tested all 22 mutants with reduced swimming and swarming motility for expression of flagellins on the bacterial surface by evaluating the amount of FliC and FljB in the sheared fraction of surface proteins by Western analysis.

**Figure 3:**
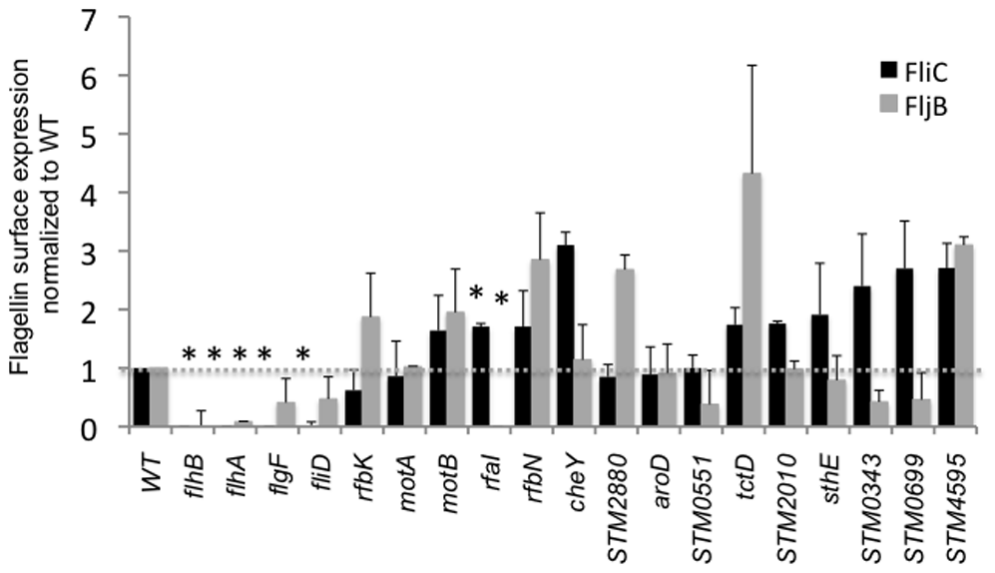
Reduced motility on semi-solid media in some mutants correlated with reduced expression of flagellin on the bacterial surface. Flagellins were sheared from bacterial surface, analyzed by Western blotting with antibodies to FliC (black bars) and FljB (grey bars) and blots were quantified by densitometry using Quantity One software. Surface expression of flagellins was normalized to the level on the surface of the wild type strain. Samples for each mutant were prepared in three independent experiments.

Mutants in *flgF*, *flgG*, and *rfbK*, genes known to be important for motility [14], [22], were both non-motile and displayed less flagellins on the bacterial surface in our screen. For other mutants however, (*motB*, *cheY*) the presence of filaments is known not to be sufficient for the ability of these mutants to move from the site of inoculation on motility plates. Our mutants in *motB* and *cheY* displayed wild type levels of flagellins on the cell surface yet were unable to spread from the site of inoculation as previously reported in the literature [23], [24] (Figure 3).

Of the nine mutants with reduced ability to move from the site of inoculation on swimming and swarming agar plates in our screen that were not previously known to be needed for this ability, all had levels of FliC on the bacterial surface comparable to wild type (Figure 3). Several of the mutants we identified in this category (*STM0343, STM0551*, *STM0699)* expressed less of the minor flagellin FljB on the bacterial surface compared to wild type ATCC14028 (Figure 3, Figure S2). Two of these mutants (*STM0343* and *STM0551*) have deletions in genes encoding EAL-domains containing proteins, predicted to be involved in the metabolism of cyclic diguanosine monophosphate (c-di-GMP) [25], . Purified STM0551 possesses phosphodiesterase activity *in vitro* that is abolished by a point mutation in the EAL domain [25]. Based on the current model of c-di-GMP metabolism in bacteria, mutation in c-di-GMP phosphodiesterases results in accumulation of c-di-GMP leading to decreased expression of flagellins, and loss of motility [27]. Furthermore *STM0551* appears to be a negative regulator of type 1 fimbria. Activation of *fim* genes includes the activation of a negative regulator of motility *fimZ* [25]. Over-expression of FimZ is known to repress swimming motility in *Salmonella* [28]. In agreement with these findings, deletion of *STM0551* abrogates outward movement on both swimming and swarming agar in *Salmonella*. Thus, data from our screen is consistent with previously published work, and shows that the amount of flagellin sheared from the bacterial surface can be correlated with motility for some but not all mutants.

### Mutants with reduced swimming motility

We identified 49 mutants that had reduced movement from the site of inoculation on swimming motility plates (less than 25% of wild type swimming) but appeared to behave similarly to the wild type organism on swarming motility plates (Table 1, Table S1). We were surprised that the ability to perform these two different types of motility could be uncoupled, and we note that further microscopic study of these mutants during swarming will provide clues regarding these seemingly paradoxical phenotypes. Sixteen mutants identified in this group were known to have reduced swimming motility including *flgE*, *fliH*, *arcA*, *yhjH* and *tatC* [4], . In addition to mutants mentioned above, we identified 33 mutants that had reduced motility on swimming agar but normal movement on swarming agar that were not previously known to be involved in swimming motility (Table S3). Some mutants in this category had deletions in genes encoding proteins involved in intracellular transport and metabolism, signal transduction and regulation of transcription based on COG predictions (Clusters of Orthologous Groups of proteins) [34] (Table 1). For example, our *Δfur* mutant was severely impaired on swimming agar, but not on swarming agar. We found that deletion of *phoQ*, also resulted in reduced swimming but not swarming motility.

Mutants in a number of genes necessary for LPS biosynthesis/assembly (*rfaG, rfaI, rfaL, rfaQ, rfaP, rfbC, rfbD, rfbJ, rfbM, rfbP, yibR*) had severely impaired movement on swimming motility agar. In *E. coli,* lipopolysaccharide (LPS) biosynthesis genes are not required for swimming motility [14]. Our work shows that *Salmonella* appears to require LPS biosynthesis for movement on swimming motility plates (Table 1). This finding is not unprecedented however, as others have shown that in *Salmonella rfaP and rfaJ* and in *Pseudomonas rfaL*, are required for swimming motility [22], [35], [36].

Mutants that had reduced outward movement from the site of inoculation on swimming agar but normal swarming movement, also included deletion mutants in genes encoding important virulence factors such as fimbria (*stjC, stdD*), Type Three Secretion System components *invF, sipA, sptP* (T3SS 1), and *ssaV* (T3SS 2) as well as Type Six Secretion System (T6SS, SPI-6 encoded) components *STM0289* and *STM0295* [37], [38]. STM0289 is a member of Vrg family of proteins required for effectors delivery via T6SS. Ours is the first description of T6SS encoded on SPI-6 potential involvement in motility. Finally, fourteen mutants, the largest group of mutants that swam poorly but swarmed normally, are not annotated or do not have a previously described function or phenotype. Thus, we describe the first functional data and potential phenotypes for these genes.

### Mutants with reduced swarming motility

We identified 49 mutants with reduced ability to move outward from the site of inoculation on swarming agar (less than 25% of wild type) but with normal outward migration on swimming agar (Table S4). None of the genes that we identified in this category were directly implicated previously in the ability to swarm. A limited number of reports describe genes required for swarming but not swimming motility. Those include *E.coli recA*, *S*.Typhimurium *flhE* and *Proteus mirabilis waaL* [39]–[42] and were not present in the library used for the current study. Deletion mutants in a number of transcriptional regulators, including STM1355 and *torR* (STM3824) appear to be involved in the regulation of swarming motility in *Salmonella*.

Several of these non-swarming mutants had deletions in genes involved in energy production/conversion and transport/metabolism as predicted by COG assignments (12 out of 49, Table 1). For example, mutants in *STM0762*, a gene that encodes fumarase, a TCA cycle enzyme involved in energy production, cannot swarm. Several additional mutants, *ΔSTM0849, ΔSTM0056 and ΔSTM1258,* have putative functions in transport or energy production. *STM0849* is a homolog of *yliB*, a ppGpp-dependent glutathione importer encoded on *yliABCD* operon [43]–[45] and *STM0056* is annotated as a putative oxalacetate decarboxylase gamma subunit [46]. Our results are consistent with previous proteomic approaches demonstrating that genes associated with energy production and *de novo* synthesis are required for swarming [47] and with the current thinking that swarming is an energetically costly process.

We identified a mutant in *srfC as* poor swarmer but normal swimmer. This gene is clustered with the flagellar class 2 genes and was determined to be under FlhDC control [48]. A polar mutation in an upstream gene in the *srfABC* operon, *srfB*, or deletion of the whole operon affected only swarming motility [49]. Similar to recent reports on swarming motility of *E. coli*, *Pseudomonas aeruginosa* and *Xenorhabdus nematophila* [14], [50], [51], mutations in fimbrial genes, *pefC* and *pefD* (encoding for usher and chaperone proteins, respectively) and in *lpfE* (long polar fimbrial minor protein) strongly affected swarming motility with no significant effect on swimming motility.

Interestingly, we found that a *fliB* mutant was defective in swarming, but not in swimming. FliB methylates the lysine residues of flagellin [52]. Previous studies showed that the loss of *fliB* did not affect swimming motility and it is thought that flagellin methylation by FliB is required for *Salmonella* virulence but not for flagellin function [48]. Our data suggest that flagellin methylation is required for swarming motility.

Swarming and virulence are linked in several bacteria [53]. 21 out of 49 of our mutants with defects in swarming are deleted for genes associated with virulence. *STM1131, STM2303, STM4030.S, STM4262* (*siiF*) are reported to be needed for full virulence in mice and calves [15], [54], [55] and mutants in these genes have reduced swarming in our assays. *pagC*, *mgtB* and *STM0859* also had reduced motility in our assays and are part of the *phoPQ* regulon in *Salmonella* [56]–[58]. Finally, swarming motility was also compromised in mutants that had deletions of genes encoded on SPI-14 (*STM0859*), a region important for virulence in chickens [59]. STM0859 was recently reported to be co-regulated with the type three secretion system encoded on SPI-2 [60].

We identified a group of genes that when deleted reduce the ability to swarm to less than 25% of the ability of the wild type organism without significant reductions in the ability to move away from the site of inoculation on swimming motility plates. Some of the products of these genes may be involved in generating movement, signaling when conditions exist for swarming, surface properties or other qualities that are directly involved in the ability to swarm, while others may affect swarming indirectly. Careful quantification and microscopic examination of each of these mutants will be useful to fit the corresponding genes into an overall framework with respect to their roles in swarming motility.

### The identification of hypermotile mutants

**Figure 4:**
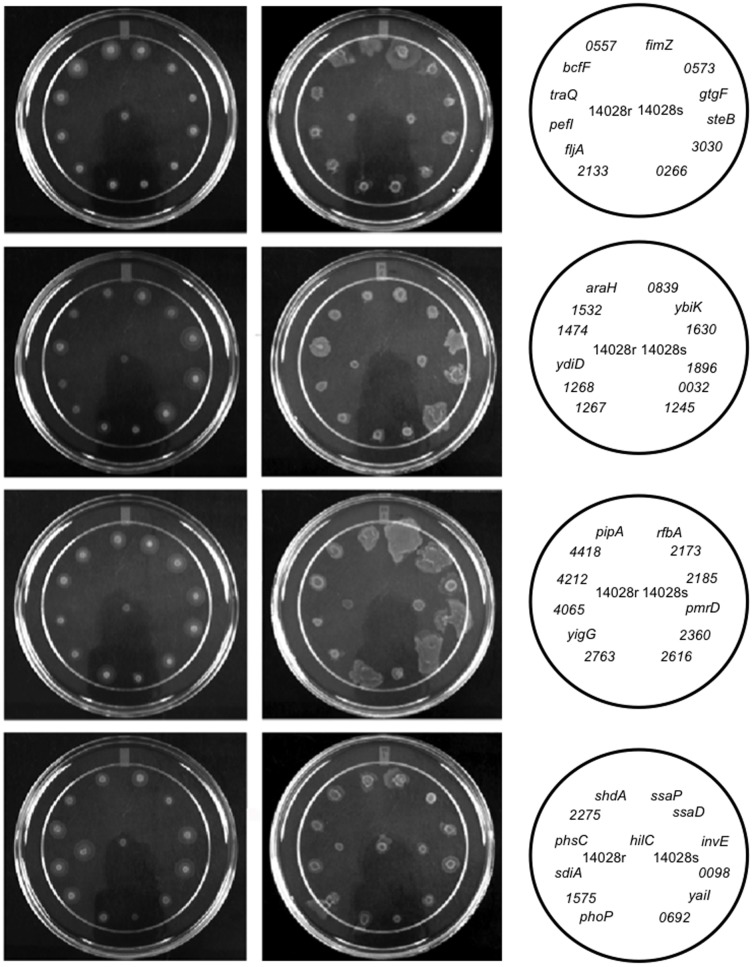
Confirmation of hypermotility phenotypes. Stationary cultures normalized by OD_600_ were spotted on swimming or swarming agar along with wild type strain, ATCC14028s. Rough strain, ATCC 14028r, was used as a negative control on swarming agar. After 3.5 and 5 hours of incubation at 37°C on swimming or swarming plates, respectively, the diameter of each bacterial growth area was measured in three independent experiments, with each experiment performed in triplicate.

As we were using 96-well format to screen our deletion mutant library for motility phenotypes, multiple observations of each screening plate were required in order to identify phenotypes before colonies intersected obscuring individual phenotypes. Early and frequent observation allowed us to identify mutants that had increased ability to move away from the site of inoculation relative to the wild type organism. We observed thirty-nine mutants to have a larger swimming and/or swarming ring diameter as compared to the wild type observed on the same plate at the same time point post inoculation (Figure 4). Of this group, we found 14 mutants that had generalized improvement in motility, 17 mutants with increased swimming motility, and 8 mutants with increased swarming motility compared to wild type (Table 2). Movies S1, S2, and S3 show examples of mutants with increased swarming motility, able to move across swarming agar faster than wild type cells (See Supporting Information Legend).

**Table 2:** Mutants with enhanced motility.

STM	Gene	Motility compared to wild type*	
		Swimming Mean ± SD	Swarming Mean ± SD
*STM0026*	*bcfF*	**1.32±0.26** **	**2.55±0.64**
*STM0032*		**1.49±0.24**	1.42**±**0.75
*STM0098*		**1.65±0.37**	**1.46±0.29**
*STM0266*		1.13**±**0.35	**1.59±0.45**
*STM0387*	*yaiI*	**1.42±0.23**	1.15**±**0.25
*STM0549*	*fimZ*	**1.32±0.20**	1.89**±**1.13
*STM0557*	*gtrC*	**1.38±0.24**	**2.13±0.52**
*STM0573*		1.14**±**0.17	**1.64±0.32**
*STM0839*		**1.20±0.20**	**1.57±0.67**
*STM0847*	*ybiK*	**1.53±0.49**	**1.36±0.29**
*STM1087*	*pipA*	**1.30±0.22**	**1.50±0.35**
*STM1231*	*phoP*	**1.39±0.34**	**1.69±0.56**
*STM1344*	*ydiV*	**1.29±0.16**	0.93**±**0.18
*STM1350*	*ydiD*	**1.61±0.79**	1.20**±**0.63
*STM1395*	*ssaD*	**1.41±0.30**	0.93**±**0.24
*STM1417*	*ssaP*	**1.47±0.27**	1.25**±**0.64
*STM1575*		**1.53±0.19**	1.35**±**0.40
*STM1629*	*steB*	0.98**±**0.26	**1.71±0.33**
*STM1630*		**1.72±0.37**	**1.62±0.50**
*STM1896*		**1.50±0.21**	**1.94±1.01**
*STM1950*	*sdiA*	**1.60±0.26**	**1.49±0.45**
*STM2063*	*phsC*	**1.46±0.27**	1.23**±**0.30
*STM2095*	*rfbA*	**1.41±0.26**	2.08**±**1.11
*STM2133*		1.08**±**0.10	**1.55±0.46**
*STM2173*		**1.41±0.22**	**1.62±0.70**
*STM2185*		**1.62±0.22**	**1.49±0.56**
*STM2304*	*pmrD*	**1.49±0.23**	**1.71±0.44**
*STM2360*		**1.35±0.16**	1.43**±**0.74
*STM2513*	*shdA*	**1.37±0.23**	1.14**±**0.26
*STM2616*		**1.31±0.28**	0.90**±**0.09
*STM2763*		**1.68±0.35**	1.52**±**0.79
*STM2770*	*fljA*	1.05**±**0.08	**1.39±0.26**
*STM2867*	*hilC*	**1.67±0.26**	**1.58±0.34**
*STM2897*	*invE*	**1.56±0.30**	1.41**±**0.51
*STM3954*	*yigG*	**1.36±0.22**	1.19**±**0.33
*STM4212*		1.14**±**0.24	**1.47±0.45**
*STM4418*		**1.26±0.21**	1.40**±**0.68
*PSLT013*	*pefI*	0.98**±**0.19	**1.18±0.11**
*PSLT098*	*traQ*	1.04**±**0.22	**1.33±0.23**

* - Diameter of swimming and swarming rings were measured and compared to wild type. Results are shown as the mean of six independent experiments.

** - Bold indicates statistical significance, *p*<0.05.

Hypermotility phenotypes have not been well described in *Salmonella*, and there are only a limited number of reports describing hypermotility as a phenotype in bacteria, primarily in *Pseudomonas aeruginosa* and *Proteus mirabilis* [61]–[69] (Table S5). Thus, there is little existing data to easily validate and benchmark the hypermotility phenotypes we observed, yet a few mutations are known to promote hypermotility. First, over-expression of FimZ, a positive regulator of Type I fimbriae and negative regulator of flagellar motility, represses swimming motility in *Salmonella* [28]. A *fimZ* deletion mutant is expected to be hypermotile, and consistent with this prediction our deletion mutant in *fimZ* was indeed hypermotile (Figure 4 and Table 2).

Second, deletion of *STM1344* (*ydiV*), annotated by COG database as a gene involved in signal transduction, resulted in improved swimming motility in our assays (Table 2). YdiV negatively regulates motility via binding to the FlhD_4_C_2_ complex to prevent interaction of this complex with DNA [70]. YdiV also functions as an adaptor protein that binds FlhD and delivers FlhD_4_C_2_ to ClpXP protease for proteolytic degradation [71] and connects flagellar gene expression to nutrient starvation [72]. In agreement with previously reported role of EAL-containing protein YdiV in negative regulation of motility in uropathogenic *E. coli* [73] and *S*. Typhimurium [74], we found that *ydiV* deletion mutant in *S*. Typhimurium was more motile on swimming agar.

**Figure 5:**
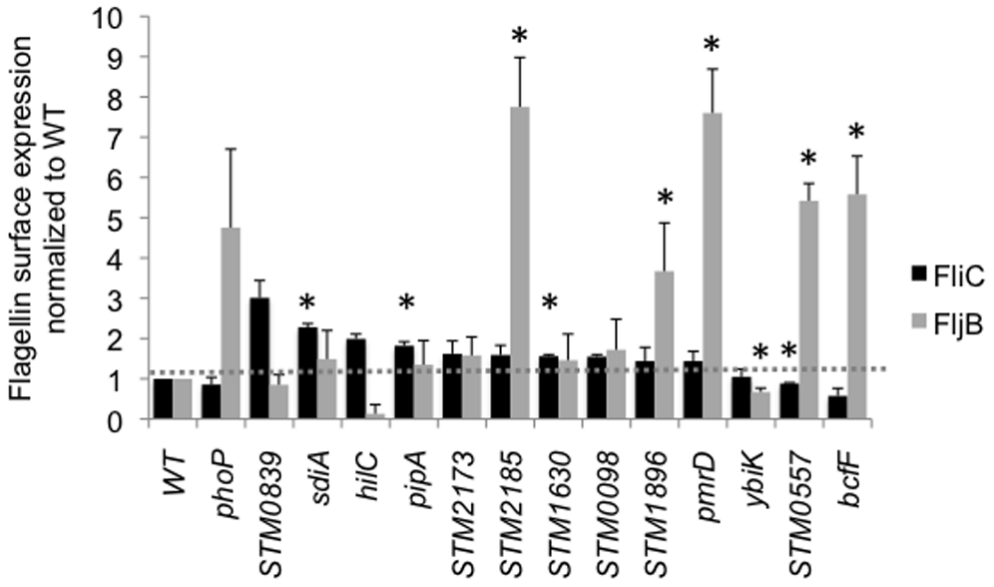
Elevated levels of flagellin were present on the bacterial surface of some mutants with improved swimming and swarming motility. Flagellins were sheared from bacterial surface, analyzed by Western blotting with antibodies to FliC and FljB and blots were quantified by densitometry using Quantity One software. Surface expression of flagellins was normalized to the level on the surface of the wild type strain. Samples for each mutant were prepared in three independent experiments.

Third, in our screen a *ΔphoP* mutant displayed increased motility both on swimming and swarming plates (Table 2, Figure 4). PhoP, the response regulator DNA-binding protein of PhoP/PhoQ two component system [75], [76], negatively regulates motility in various microorganisms including *Photorhabdus luminescens, Pseudomonas aeruginosa*, *Proteus mirabilis* and in uropathogenic *E. coli*, where a *phoP* nulls are hypermotile [66], [77]–[79]. Moreover, null allele in *Proteus mirabilis phoP* homolog exhibits an elevated level of *flhDC* [66]. Finally, in uropathogenic *E. coli* inactivation of *phoP* results in the increased expression of flagellin on the bacterial surface [78]. Similar to uropathogenic *E. coli phoP* mutant in *S*. Typhimurium increased motility correlated with increased flagellin expression on the bacterial surface (Figure 5).

We hypothesized that enhanced motility of at least some mutants could be due to increased expression of flagellins on the bacterial cell surface as was previously shown for *phoP* mutant in uropathogenic *E. coli* [78] and for *ydiV* mutant in *Salmonella* and uropathogenic *E. coli* [73], [80]. Therefore, we examined the amount of FliC and FljB on the bacterial surface for each mutant that displayed hypermotility both on swimming and swarming agar (Figure 5, Figure S2). We found that while our *ΔphoP* mutant had wild type level of expression for FliC, it also had more FljB on the bacterial surface than the isogenic wild type organism. Furthermore, our examination of flagellar proteins sheared from the bacterial surface showed that thirteen out of fourteen of our hypermotile mutants had more FliC or FljB on the bacterial surface (Figure 5, Figure S2) than the wild type. It seems plausible that the hypermotility phenotype we observed for this highly diverse group of mutants could be the result of up-regulation of flagella.

Interestingly, mutations in several fimbrial operons also result in hypermotility phenotypes, *ΔSTM0026* (*bcfF*) mutants were hypermotile and the *bcfABCDEFG* (bovine colonization factor) operon has previously been implicated in virulence in mice [81]. Inactivation of *bcfF* improves biofilm formation on HEp-2 tissue culture cells and chicken intestinal epithelium in comparison to wild type [82]. Mutants in FimZ (STM0549) were also hypermotile. Inactivation of FimZ reduces expression from the P*_fimA_* promoter and prevents serovar Typhimurium from making type I fimbriae [83], [84]. FimZ also binds to the P*_flhDC_* promoter and represses the expression of *flhDC* operon.

### Concluding remarks

In this study we evaluated the ability of 1023 defined non-polar single gene deletions of *Salmonella* Typhimurium to move away from the site of inoculation on swimming and swarming agar plates. Over 90% of deletions in our collection were introduced in genes present exclusively in *Salmonella* or closely related pathogenic bacteria from the *Enterobacteriaceae*. Many of the mutants used in this study did not have any functions previously assigned. We confirmed motility phenotypes associated with loss of genes involved in flagellar regulon, LPS biosynthesis and chemotaxis. We identified a number of novel contributors to bacterial motility including both known and uncharacterized genes in our pathogenicity-biased collection. Furthermore, we found that there are genes needed for both types motility, and there are genes that make a unique contribution to different kinds of motility. Finally, we identified mutations in a number of genes result in increased motility. Determination of the molecular mechanisms of the improved motility is a fascinating area of the future work.

## Supporting Information

Figure S1 **Mutants with severe defects in swimming and swarming motility grow indistinguishably from wild type.** Overnight cultures were subcultured at 1/100 ration in LB-broth and incubated at 37°C with shaking. Bacterial growth was monitored by OD600 in three independent experiments.
(TIF)

Figure S2 **Flagellin expression on the cell surface correlates with the ability to move on swimming and swarming agar for some, but not all mutants.** Flagellins sheared from the bacterial surface from strains with decreased (A) or increased (B, C) motility grown in LB-broth were analyzed by Western blotting with µ-FliC and µ-FljB sera. The whole cell lysates for each sample were also analyzed by SDS-PAGE and stained with Coomassie as a loading control.
(TIF)

Table S1 **Swimming and swarming motility scores from high-throughput screening.**
(XLS)

Table S2 **Confirmation of motility defects (loss of>75% motility compared to wild type) in mutants identified in primary screening.**
(DOCX)

Table S3 **Mutants with defect in swimming motility only.**
(DOCX)

Table S4 **Mutants with defect in swarming motility.**
(DOCX)

Table S5 **Previously described hypermotility in bacteria.**
(DOCX)

Table S6 **Densitometry data underlying** **Figures 3** **and** **5** **, and representative images of Western blots used to generate this data.**
(XLSX)

Movie S1 **WT_400x_6h.mov shows examples of the swarming motility of the wild type organism.**
(MP4)

Movie S2 **fimZ_400x_6h.mov shows the swarming motility of a deletion mutant in fimZ that has increased swarming motility relative to the wild type.**
(MP4)

Movie S3 **STM1630_400x_6h.mov shows the swarming motility of a deletion mutant in STM1630 that has increased swariming motility relative to the wild type.**
(MP4)
