# Supplementary figures and images for: Identification of Novel Factors Involved in Modulating Motility of Salmonella enterica Serotype Typhimurium
Source: PLoS One. 2014 Nov 4;9(11):e111513. doi: 10.1371/journal.pone.0111513 (PMC4219756; doi:10.1371/journal.pone.0111513)

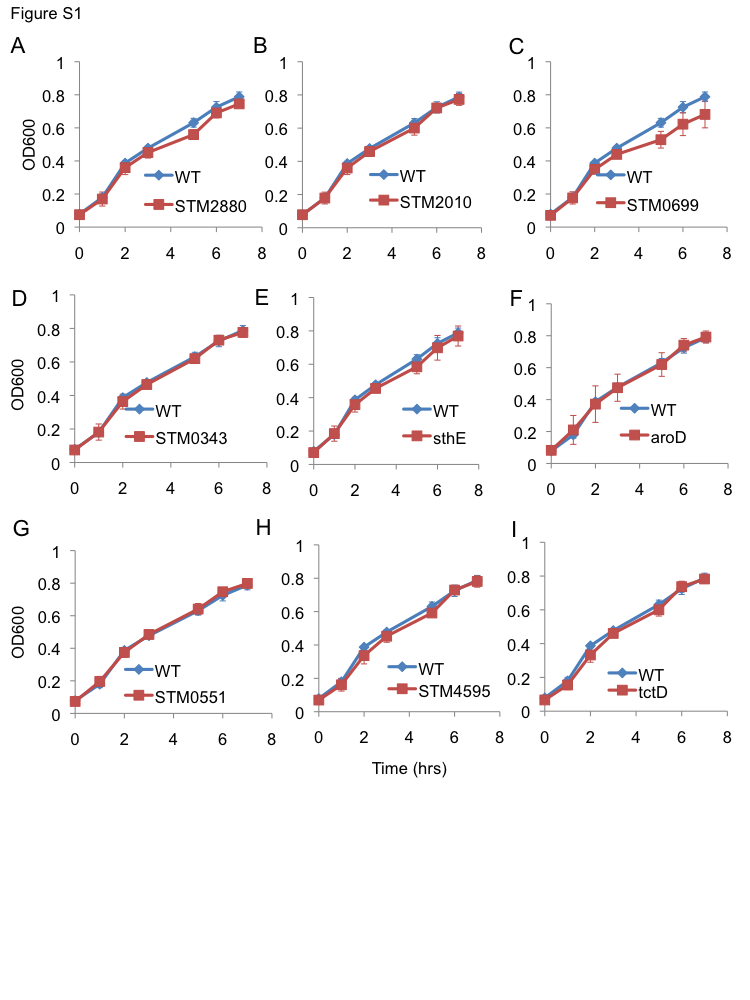

Supplement: Figure S1 — Mutants with severe defects in swimming and swarming motility grow indistinguishably from wild type. Overnight cultures were subcultured at 1/100 ration in LB-broth and incubated at 37°C with shaking. Bacterial growth was monitored by OD600 in three independent experiments. (TIF) [file pone.0111513.s001.tif]

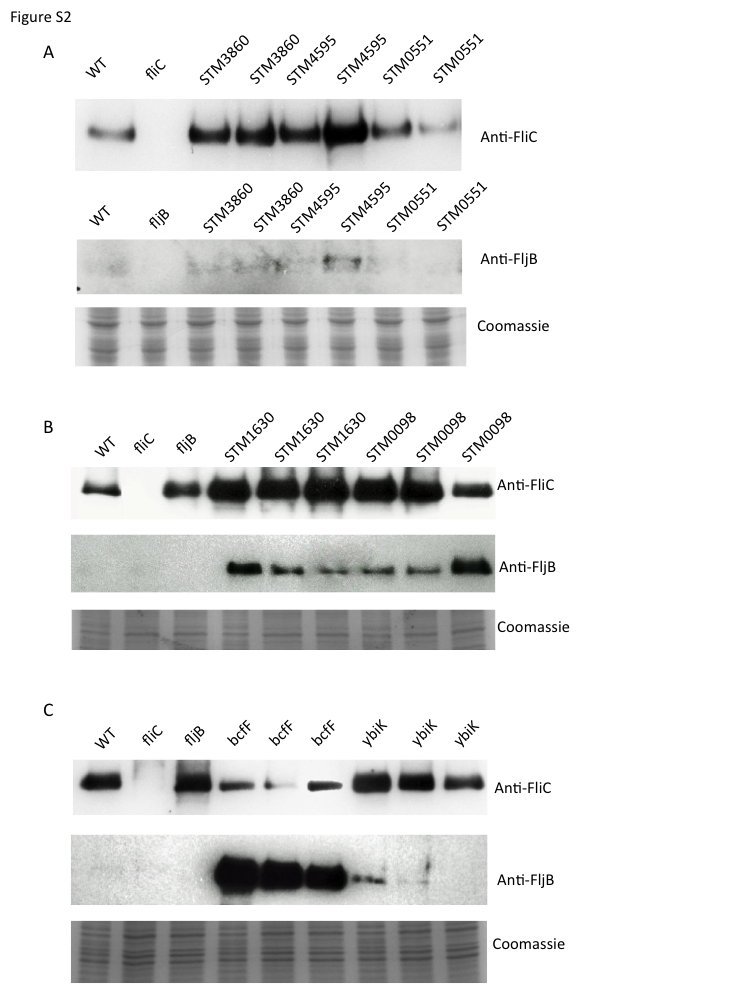

Supplement: Figure S2 — Flagellin expression on the cell surface correlates with the ability to move on swimming and swarming agar for some, but not all mutants. Flagellins sheared from the bacterial surface from strains with decreased (A) or increased (B, C) motility grown in LB-broth were analyzed by Western blotting with µ-FliC and µ-FljB sera. The whole cell lysates for each sample were also analyzed by SDS-PAGE and stained with Coomassie as a loading control. (TIF) [file pone.0111513.s002.tif]
