# Supplementary material for: Identification of Novel Factors Involved in Modulating Motility of Salmonella enterica Serotype Typhimurium
Source: PLoS One. 2014 Nov 4;9(11):e111513. doi: 10.1371/journal.pone.0111513 (PMC4219756; doi:10.1371/journal.pone.0111513)
Supplement: Table S2 — Confirmation of motility defects (loss of>75% motility compared to wild type) in mutants identified in primary screening. (DOCX) [file pone.0111513.s004.docx]

**Table S2. Confirmation of motility defects (loss of >75% motility compared to wild type) in mutants identified in primary screening.**

| STM | Gene | Motility compared to wild type* | |
| --- | --- | --- | --- |
|  |  | Swimming Mean ± SD | Swarming Mean ± SD |
| ***STM1178*** | *flgF* | **0.00 ± 0.00**** | **0.01 ± 0.01** |
| ***STM1179*** | *flgG* | **0.00 ± 0.00** | **0.01 ± 0.01** |
| ***STM1913*** | *flhA* | **0.00 ± 0.00** | **0.01 ± 0.01** |
| ***STM1914*** | *flhB* | **0.00 ± 0.00** | **0.00 ± 0.00** |
| ***STM1916*** | *cheY* | **0.23 ± 0.04** | **0.01 ± 0.01** |
| ***STM1922*** | *motB* | **0.00 ± 0.00** | **0.01 ± 0.01** |
| ***STM1923*** | *motA* | **0.00 ± 0.00** | **0.00 ± 0.00** |
| ***STM1960*** | *fliD* | **0.14 ± 0.01** | **0.05 ± 0.05** |
| ***STM1976*** | *fliM* | **0.02 ± 0.02** | **0.05 ± 0.05** |
| ***STM2083*** | *rfbK* | **0.04 ± 0.05** | **0.02 ± 0.03** |
| ***STM2085*** | *rfbN* | **0.12 ± 0.08** | **0.02 ± 0.01** |
| ***STM3718*** | *rfaI* | **0.17 ± 0.11** | **0.02 ± 0.01** |
| ***STM0343*** |  | **0.20 ± 0.04** | **0.08 ± 0.06** |
| *STM0363* |  | 0.78 ± 0.22 | 0.50 ± 0.10 |
| ***STM0551*** |  | **0.05 ± 0.02** | **0.03 ± 0.02** |
| ***STM0669*** |  | **0.23 ± 0.10** | **0.00 ± 0.00** |
| ***STM1358*** | *aroD* | **0.11 ± 0.05** | **0.05 ± 0.05** |
| *STM1573* |  | 0.38 ± 0.11 | 0.00 ± 0.00 |
| *STM1660* | *fnr* | 0.49 ± 0.11 | 0.43 ± 0.12 |
| *STM1829* |  | 0.35 ± 0.07 | 0.06 ± 0.06 |
| *STM1987* |  | 0.33 ± 0.05 | 0.00 ± 0.00 |
| ***STM2010*** |  | **0.00 ± 0.00** | **0.00 ± 0.00** |
| *STM2330* | *lrhA* | 0.99 ± 0.10 | 1.04 ± 0.22 |
| *STM2341* |  | 0.43 ± 0.09 | 0.05 ± 0.05 |
| *STM2342* |  | 0.46 ± 0.07 | 0.05 ± 0.05 |
| *STM2435* | *prxK* | 0.41 ± 0.11 | 0.34 ± 0.28 |
| ***STM2785*** | *tctD* | **0.11 ± 0.04** | **0.02 ± 0.02** |
| ***STM2880*** |  | **0.18 ± 0.03** | **0.02 ± 0.02** |
| *STM2901* |  | 0.41 ± 0.08 | 0.24 ± 0.09 |
| *STM3501* | *envZ* | 0.43 ± 0.03 | 0.01 ± 0.01 |
| *STM3737* |  | 0.48 ± 0.11 | 0.02 ± 0.02 |
| *STM3860* |  | 0.51 ± 0.05 | 0.19 ± 0.13 |
| *STM4302* |  | 0.49 ± 0.04 | 0.05 ± 0.05 |
| ***STM4591*** | *sthE* | **0.23 ± 0.01** | 0.04 ± 0.14 |
| ***STM4595*** |  | **0.21 ± 0.03** | **0.25 ± 0.22** |

* - Diameter of swimming and swarming rings were measured after 3.5 hrs and 5.5 hrs of incubation at 37 ^0^C, respectively, and compared to wild type. Results are shown as the mean of three independent experiments

** - Mutants in bold were selected for further characterization
