# Supplementary material for: Identification of Novel Factors Involved in Modulating Motility of Salmonella enterica Serotype Typhimurium
Source: PLoS One. 2014 Nov 4;9(11):e111513. doi: 10.1371/journal.pone.0111513 (PMC4219756; doi:10.1371/journal.pone.0111513)
Supplement: Table S3 — Mutants with defect in swimming motility only. (DOCX) [file pone.0111513.s005.docx]

**Table S3. Mutants with defect in swimming motility only**

| Biological processes | Gene | Known phenotype associated with motility | Reference |
| --- | --- | --- | --- |
| Motility | ***flgE*** | Mutation in *E.coli* homolog leads to defect in swimming as well as in swarming motility | (1, 2) |
|  | ***fliH*** |  |  |
| LPS biosynthesis and processing | ***rfbP*** | In *E.coli* LPS mutants we defective in swarming but not in swimming motility | (1) |
|  | ***rfbM*** |  |  |
|  | ***rfbJ*** |  |  |
|  | ***rfbC*** |  |  |
|  | ***rfbD*** |  |  |
|  | ***rfaJ*** |  |  |
|  | ***rfaG*** |  |  |
|  | ***rfaQ*** |  |  |
|  | ***rfaL*** |  |  |
|  | ***rfaP*** |  |  |
|  | *yibR* |  |  |
| Signal transduction | *phoQ* | Mutation in *E.coli* homolog resulted in defect in swarming motility | (1) |
|  | ***yhjH*** | Mutations in *E.coli* homolog as well as in *Salmonella* lead to defect in swimming but not swarming motility | (2-5) |
|  | *yjcC* |  |  |
| Energy production transport and metabolism | *STM0722* |  |  |
|  | *STM4424* |  |  |
|  | *aroA* |  |  |
|  | *fur* | Fur is a positive regulator of *flhDC* operon | (6) |
|  | *yfeJ* |  |  |
|  | *pdxK* |  |  |
|  | ***tatC*** | TatC was shown to be important for swimming but not swarming motility in *E.coli* as well as in *Salmonella* | (2, 7) |
| Transcription | *invF* |  |  |
|  | *STM2912* |  |  |
|  | *STM3696* |  |  |
|  | *STM4417* |  |  |
|  | ***arcA*** | Mutation in *E.coli* homolog resulted in defect in swimming motility | (8) |
| Virulence | *stjC* |  | (9) |
|  | *STM3026 (stdD)* |  | (10) |
|  | *sipA* |  | (11, 12) |
|  | *sptP* |  | (13) |
|  | *ssaV* |  | (14) |
|  | *ssaG* |  |  |
|  | *STM0289* |  |  |
|  | *STM0295* |  |  |
|  | *STM2743* |  | (10) |
| Others | *STM1005*  *valS*  *STM0660*  *STM0971*  *STM1040*  *STM1331*  *STM2374 sspA*  *STM3783*  *STM4216*  *STM4219*  *sRNA candidate C1023* |  |  |

*Mutants with previously known motility phenotype are shown in bold

References

1. **Inoue, T., R. Shingaki, S. Hirose, K. Waki, H. Mori, and K. Fukui.** 2007. Genome-wide screening of genes required for swarming motility in Escherichia coli K-12. Journal of Bacteriology **189:**950-957.

2. **Girgis, H. S., Y. Liu, W. S. Ryu, and S. Tavazoie.** 2007. A comprehensive genetic characterization of bacterial motility. PLoS genetics **3:**1644-1660.

3. **Simm, R., M. Morr, A. Kader, M. Nimtz, and U. Romling.** 2004. GGDEF and EAL domains inversely regulate cyclic di-GMP levels and transition from sessility to motility. Molecular microbiology **53:**1123-1134.

4. **Wang, Q., J. G. Frye, M. McClelland, and R. M. Harshey.** 2004. Gene expression patterns during swarming in Salmonella typhimurium: genes specific to surface growth and putative new motility and pathogenicity genes. Molecular microbiology **52:**169-187.

5. **Paul, K., V. Nieto, W. C. Carlquist, D. F. Blair, and R. M. Harshey.** 2010. The c-di-GMP Binding Protein YcgR Controls Flagellar Motor Direction and Speed to Affect Chemotaxis by a "Backstop Brake" Mechanism. Molecular cell.

6. **Campoy, S. J., M.; Busquets, N.; Pérez de Rozas, A.M.; Badiola, I.; Barbé, J.** 2002. Intracellular cyclic AMP concentration is decreased in Salmonella typhimurium fur mutants microbiology (Reading, England) **148:**1039-1048.

7. **Reynolds, M. M., L. Bogomolnaya, J. Guo, L. Aldrich, D. Bokhari, C. A. Santiviago, M. McClelland, and H. Andrews-Polymenis.** 2011. Abrogation of the twin arginine transport system in Salmonella enterica serovar Typhimurium leads to colonization defects during infection. PloS one **6:**e15800.

8. **Kato, Y., M. Sugiura, T. Mizuno, and H. Aiba.** 2007. Effect of the arcA mutation on the expression of flagella genes in Escherichia coli. Biosci Biotechnol Biochem **71:**77-83.

9. **Akkoç, N. Ö., B.; Tan, B.G.; Akçelik, M.** 2009. The role of stj fimbrial operon in the intestinal persistence of Salmonella Typhimurium in mice. Biologia **64:**859-863.

10. **Morgan, E., J. D. Campbell, S. C. Rowe, J. Bispham, M. P. Stevens, A. J. Bowen, P. A. Barrow, D. J. Maskell, and T. S. Wallis.** 2004. Identification of host-specific colonization factors of Salmonella enterica serovar Typhimurium. Molecular microbiology **54:**994-1010.

11. **Zhang, S. S., R.L.; Tsolis,R.M.; Stender,S.; Hardt,W.D.; Baumler,A.J.; Adams,L.G.** 2002. The Salmonella enterica serotype typhimurium effector proteins SipA, SopA, SopB, SopD, and SopE2 act in concert to induce diarrhea in calves. Infection and immunity **70:**3843-3855.

12. **Raffatellu, M. W., R.P.; Chessa,D.; Andrews-Polymenis,H.; Tran,Q.T.; Lawhon,S.; Khare,S.; Adams,L.G.; Baumler,A.J.** 2005. SipA, SopA, SopB, SopD, and SopE2 contribute to Salmonella enterica serotype typhimurium invasion of epithelial cells Infection and immunity **73:**146-154.

13. **Marcus S.L., B. J. H., Pfeifer C.G., and Finlay B.B. .** 2000. Salmonella pathogenicity islands: Big virulence in small packages. . microbes and Infection **2:**145-156.

14. **Vazquez-Torres, A. X., Y.; Jones-Carson,J.; Holden,D.W.; Lucia,S.M.; Dinauer,M.C.; Mastroeni,P.; Fang,F.C.** 2000. Salmonella pathogenicity island 2-dependent evasion of the phagocyte NADPH oxidase. Science (New York, N.Y.) **287:**1655-1658.
