# Supplementary material for: Identification of Novel Factors Involved in Modulating Motility of Salmonella enterica Serotype Typhimurium
Source: PLoS One. 2014 Nov 4;9(11):e111513. doi: 10.1371/journal.pone.0111513 (PMC4219756; doi:10.1371/journal.pone.0111513)
Supplement: Table S4 — Mutants with defect in swarming motility. (DOCX) [file pone.0111513.s006.docx]

**Table S4. Mutants with defect in swarming motility**

| Biological processes | Gene | Reference |
| --- | --- | --- |
| Signal transduction | *yciR* (encoding GGDEF-EAL domain containing protein) | (1, 2) |
| Energy production, transport and metabolism | *STM0860, STM3780,yliB, STM1635, sfbA, , sodA, STM0163, STM1546, STM0857 STM0762, STM0858* | (3-12) |
| Transcription | *ydiP, torR, rtsA (STM4315)* | (13-16) |
| Virulence | *STM1131, STM4030.S, STM4262, pagC, mgtB,* *srfC, STM1632, lpfE, STM0859, ssaU, pefD, pefC, STM2303, STM4599, STM4529, STM1258, STM1543* | (10, 17-31) |
| Others | *STM0056, STM0362, STM1856, STM1254 STM1552, STM1861, STM1926, STM1958 (fliB), STM2508, STM2594,* *ygaU, STM3125, STM3944, STM4197, STM4204, STM4574, invR* | (27, 32, 33) |

References

1. **Garcia B., L. C., Solano C., Garcia-del Portillo F., Gamazo C., and Lasa I.** 2004. Role of the GGDEF protein family in Salmonella cellulose biosynthesis and biofilm formation. Molecular microbiology **54:**264-277.

2. **Simm R., L. A., Kader A., Andersson M., and Romling U.** 2007. Role of EAL-containing proteins in multicellular behavior of Salmonella enterica serovar Typhimurium. Journal of Bacteriology **189:**3613-3623.

3. **Tsolis R.M., B. A. J., and Heffron F.** 1995. Role of Salmonella typhimurium mn-superoxide dismutase (SodA) in protection against early killing by J774 macrophages Infection and immunity **63:**1739-1744.

4. **Pattery T., H. J., and De Greve H.** 1999. Identification and molecular characterization of a novel Salmonella enteritidis pathogenicity islet encoding an ABC transporter. Molecular microbiology **33:**791-805.

5. **Eriksson S., L. S., Thompson A., Rhen M., and Hinton J.C.** 2003. Unravelling the biology of macrophage infection by gene expression profiling of intracellular Salmonella enterica. Molecular microbiology **47:**103-118.

6. **Kim, W., and M. G. Surette.** 2004. Metabolic differentiation in actively swarming Salmonella. Molecular microbiology **54:**702-714.

7. **Suzuki, H., T. Koyanagi, S. Izuka, A. Onishi, and H. Kumagai.** 2005. The yliA, -B, -C, and -D genes of Escherichia coli K-12 encode a novel glutathione importer with an ATP-binding cassette. Journal of Bacteriology **187:**5861-5867.

8. **Hautefort, I. T., A.; Eriksson-Ygberg,S.; Parker,M.L.; Lucchini,S.; Danino,V.; Bongaerts,R.J.M.; Ahmad,N.; Rhen,M.; Hinton,J.C.D.** 2008. During infection of epithelial cells Salmonella enterica serovar Typhimurium undergoes a time-dependent transcriptional adaptation that results in simultaneous expression of three type 3 secretion systems. Cellular microbiology **10:**958-984.

9. **Traxler, M. F., S. M. Summers, H.-T. Nguyen, V. M. Zacharia, G. A. Hightower, J. T. Smith, and T. Conway.** 2008. The global, ppGpp-mediated stringent response to amino acid starvation in Escherichia coli. Molecular microbiology **68:**1128-1148.

10. **Yoon, H., J. E. McDermott, S. Porwollik, M. McClelland, and F. Heffron.** 2009. Coordinated regulation of virulence during systemic infection of Salmonella enterica serovar Typhimurium. PLoS pathogens **5:**e1000306.

11. **Haneda, T., M. Sugimoto, Y. Yoshida-Ohta, Y. Kodera, M. Oh-Ishi, T. Maeda, S. Shimizu-Izumi, T. Miki, Y. Kumagai, H. Danbara, and N. Okada.** 2010. Comparative proteomic analysis of Salmonella enterica serovar Typhimurium ppGpp-deficient mutant to identify a novel virulence protein required for intracellular survival in macrophages. BMC Microbiology **10:**324.

12. **Troxell B., F. R., Porwollik S., McClelland M., and Hassan H.** 2011. The fur regulon in anaerobically grown Salmonella enterica sv. Typhimurium: Identification of new fur targets. BMC microbiology **11**.

13. **Morgan-Kiss R.M., a. C. J. E.** 2004. The Escherichia coli fadK (ydiD) gene encodes an anerobically regulated short chain acyl-CoA synthetase. The Journal of Biological chemistry **279:**37324-37333.

14. **Ellermeier C.D., E. J. R., and Slauch J.M.** 2005. HilD, HilC and RtsA constitute a feed forward loop that controls expression of the SPI1 type three secretion system regulator hilA in Salmonella enterica serovar Typhimurium. Molecular microbiology **57:**691-705.

15. **Baraquet C., T. L., Guiral M., Lafitte D., Mejean V., and Jourlin-Castelli C.** 2006. TorT, a member of a new periplasmic binding protein family, triggers induction of the tor respiratory system upon trimethylamine N-oxide electron-acceptor binding in Escherichia coli The Journal of Biological chemistry **281:**38189-38199.

16. **Pullinger G.D., v. D. P. M., Dziva F., and Stevens M.P.** 2010. Role of two-component sensory systems of Salmonella enterica serovar Dublin in the pathogenesis of systemic salmonellosis in cattle. Microbiology (Reading, England) **156:**3108-3122.

17. **Chaudhuri, R. R., S. E. Peters, S. J. Pleasance, H. Northen, C. Willers, G. K. Paterson, D. B. Cone, A. G. Allen, P. J. Owen, G. Shalom, D. J. Stekel, I. G. Charles, and D. J. Maskell.** 2009. Comprehensive Identification of Salmonella enterica Serovar Typhimurium Genes Required for Infection of BALB/c Mice. PLoS Pathogens **5:**e1000529.

18. **Wang, Q., S. Mariconda, A. Suzuki, M. McClelland, and R. Harshey.** 2006. Uncovering a large set of genes that affect surface motility in Salmonella enterica serovar Typhimurium. Journal of Bacteriology **188:**7981-7984.

19. **Morgan, E., J. D. Campbell, S. C. Rowe, J. Bispham, M. P. Stevens, A. J. Bowen, P. A. Barrow, D. J. Maskell, and T. S. Wallis.** 2004. Identification of host-specific colonization factors of Salmonella enterica serovar Typhimurium. Molecular microbiology **54:**994-1010.

20. **Snavely M.D., M. C. G., and Maguire M.E.** 1991. The mgtB Mg2+ transport locus of Salmonella typhimurium encodes a P-type ATPase. The Journal of Biological chemistry **266:**815-823.

21. **Baumler A.J., T. R. M., Bowe F.A., Kusters J.G., Hoffmann S., and Heffron F.** 1996. The pef fimbrial operon of Salmonella typhimurium mediates adhesion to murine small intestine and is necessary for fluid accumulation in the infant mouse. Infection and immunity **64:**61-68.

22. **Blanc-Potard A.B., S. F., Kayser J., and Groisman E.A.** 1999. The SPI-3 pathogenicity island of Salmonella enterica. J Bacteriol **181:**998-1004.

23. **Waterman S.R., a. H. D. W.** 2003. Functions and effectors of the Salmonella pathogenicity island 2 type III secretion system. Cellular microbiology **5:**501-511.

24. **Monsieurs, P., S. Keersmaecker, W. W. Navarre, M. W. Bader, F. Smet, M. McClelland, F. C. Fang, B. Moor, J. Vanderleyden, and K. Marchal.** 2005. Comparison of the PhoPQ Regulon in Escherichia coli and Salmonella typhimurium. Journal of Molecular Evolution **60:**462-474.

25. **Navarre, W. W., T. A. Halsey, D. Walthers, J. Frye, M. McClelland, J. L. Potter, L. J. Kenney, J. S. Gunn, F. C. Fang, and S. J. Libby.** 2005. Co-regulation of Salmonella enterica genes required for virulence and resistance to antimicrobial peptides by SlyA and PhoP/PhoQ. Molecular microbiology **56:**492-508.

26. **Shah, D. H., M. J. Lee, J. H. Park, J. H. Lee, S. K. Eo, J. T. Kwon, and J. S. Chae.** 2005. Identification of Salmonella gallinarum virulence genes in a chicken infection model using PCR-based signature-tagged mutagenesis. Microbiology (Reading, England) **151:**3957-3968.

27. **Frye, J., J. E. Karlinsey, H. R. Felise, B. Marzolf, N. Dowidar, M. McClelland, and K. T. Hughes.** 2006. Identification of new flagellar genes of Salmonella enterica serovar Typhimurium. Journal of Bacteriology **188:**2233-2243.

28. **Groisman, E. A., and C. Mouslim.** 2006. Sensing by bacterial regulatory systems in host and non-host environments. Nature Reviews Microbiology **4:**705-709.

29. **Ledeboer, N. A., J. G. Frye, M. McClelland, and B. D. Jones.** 2006. Salmonella enterica serovar Typhimurium requires the Lpf, Pef, and Tafi fimbriae for biofilm formation on HEp-2 tissue culture cells and chicken intestinal epithelium. Infection and immunity **74:**3156-3169.

30. **Morgan E., B. A. J., Carnell S.C., Wallis T.S., and Stevens M.P.** 2007. SiiE is secreted by the Salmonella enterica serovar Typhimurium pathogenicity island 4-encoded secretion system and contributes to intestinal colonization in cattle. Infection and immunity **75:**1524-1533.

31. **Santiviago, C. A., M. M. Reynolds, S. Porwollik, S. H. Choi, F. Long, H. L. Andrews-Polymenis, and M. McClelland.** 2009. Analysis of pools of targeted Salmonella deletion mutants identifies novel genes affecting fitness during competitive infection in mice. PLoS pathogens **5:**e1000477.

32. **Pfeiffer V., S. A., Tomer R., Tedin K., Brinkmann V., and Vogel J.** 2007. A small non-coding RNA of the invasion gene island (SPI-1) represses outer membrane protein synthesis from the Salmonella core genome. Molecular microbiology **66:**1174-1191.

33. **Erhardt M., a. H. K. T.** 2010. C-ring requirement in flagellar type III secretion is bypassed by FlhDC upregulation Molecular microbiology **75:**376-393.
