# Supplementary material for: Identification of Novel Factors Involved in Modulating Motility of Salmonella enterica Serotype Typhimurium
Source: PLoS One. 2014 Nov 4;9(11):e111513. doi: 10.1371/journal.pone.0111513 (PMC4219756; doi:10.1371/journal.pone.0111513)
Supplement: Table S5 — Previously described hypermotility in bacteria. (DOCX) [file pone.0111513.s007.docx]

**Table S5. Previously described hypermotility in bacteria**

| Organism | Gene | Type of motility | Homolog in S.Typhimurium | Hypermotility phenotype |
| --- | --- | --- | --- | --- |
| *Pseudomonas aeruginosa* | *PA1303* | Swarming (1) | *STM2582* | ND |
|  | *sadB* | Swarming (2) | NF | ND |
|  | *pilJ* |  | *STM3152* | No |
|  | *sadC* | Swarming (3) | *STM4551* | No |
|  | *roeA* | Swarming (4) | *STM1987* | No |
|  | *pilY1* | Swarming (5) | NF | ND |
|  | *phoP* | Swarming (6) | *phoP (STM1231)* | Swimming and swarming |
| *Vibrio fisheri* | *gacA* | Swimming (7, 8) | *sirA (STM1947)* | No |
| *Vibrio cholerae* | *toxR/toxS* | Swimming (9) | NF | ND |
| *Xenorhabdus nematophila* | *ompR* | Swarming (10) | *ompR (STM3502)* | No (11) and this paper |
| *Proteus mirabilis* | *rcsBCD* | Swarming (12-14) | *rcsB* | Swimming and swarming (15, 16) |
|  | *rppA* | Swarming (17) | *phoP (STM1231)* | Swimming and swarming |
| *Photorhabdus luminescens* | *phoP* | Swimming (18) | *phoP (STM1231)* | Swimming and swarming |
| Uropathogenic *E.coli* | *phoP* | Swimming (19) | *phoP (STM1231)* | Swimming and swarming |
|  | *ydiV* | Swimming (20) | *ydiV (STM1344)* | Swimming (15, 21) and this paper |
| *E.coli* O157:H7 | *sdiA* | Swimming (22) | *sdiA*  *(STM1950)* | Swimming and swarming |
| *E.coli* K-12 | *sdiA* | Swimming (23) | *sdiA (STM1950)* | Swimming and swarming |

NF-not found;

ND – not determined

References

1. **Waite, R. D., R. S. Rose, M. Rangarajan, J. Aduse-Opoku, A. Hashim, and M. A. Curtis.** 2012. Pseudomonas aeruginosa possesses two putative Type 1 signal peptidases, LepB and PA1303, each with distinct roles in physiology and virulence. Journal of Bacteriology.

2. **Caiazza, N. C., J. H. Merritt, K. M. Brothers, and G. A. O'Toole.** 2007. Inverse regulation of biofilm formation and swarming motility by Pseudomonas aeruginosa PA14. Journal of Bacteriology **189:**3603-3612.

3. **Merritt, J. H., K. M. Brothers, S. L. Kuchma, and G. A. O'Toole.** 2007. SadC reciprocally influences biofilm formation and swarming motility via modulation of exopolysaccharide production and flagellar function. Journal of Bacteriology **189:**8154-8164.

4. **Merritt, J. H., D. G. Ha, K. N. Cowles, W. Lu, D. K. Morales, J. Rabinowitz, Z. Gitai, and G. A. O'Toole.** 2010. Specific control of Pseudomonas aeruginosa surface-associated behaviors by two c-di-GMP diguanylate cyclases. mBio **1:**e00183-10.

5. **Kuchma, S. L., A. E. Ballok, J. H. Merritt, J. H. Hammond, W. Lu, J. D. Rabinowitz, and G. A. O'Toole.** 2010. Cyclic-di-GMP-mediated repression of swarming motility by Pseudomonas aeruginosa: the pilY1 gene and its impact on surface-associated behaviors. Journal of Bacteriology **192:**2950-2964.

6. **Brinkman, F. S., E. L. Macfarlane, P. Warrener, and R. E. Hancock.** 2001. Evolutionary relationships among virulence-associated histidine kinases. Infection and immunity **69:**5207-5211.

7. **Millikan D.S., a. R. E. G.** 2002. Alterations in Vibrio fischeri motility correlate with a delay in symbiosis initiation and are associated with additional symbiotic colonization defects. Applied and Environmental Microbiology **68:**2519-2528.

8. **Whistler C.A., a. R. E. G.** 2003. GacA regulates symbiotic colonization traits of Vibrio fischeri and facilitates a beneficial association with an animal host J Bacteriol **185:**7202-7212.

9. **Gardel C.L., a. M. J. J.** 1996. Alterations in Vibrio cholerae motility phenotypes correlate with changes in virulence factor expression Infection and immunity **64:**2246-2255.

10. **Kim D.J., B. B., George N., and Forst S.** 2003. Inactivation of ompR promotes precocious swarming and flhDC expression in Xenorhabdus nematophila. J Bacteriol **185:**5290-5294.

11. **Toguchi, A., M. Siano, M. Burkart, and R. M. Harshey.** 2000. Genetics of swarming motility in Salmonella enterica serovar Typhimurium: critical role for lipopolysaccharide. Journal of Bacteriology **182:**6308-6321.

12. **Belas, R., R. Schneider, and M. Melch.** 1998. Characterization of Proteus mirabilis precocious swarming mutants: identification of rsbA, encoding a regulator of swarming behavior. Journal of Bacteriology **180:**6126-6139.

13. **Liaw, S. J., H. C. Lai, S. W. Ho, K. T. Luh, and W. B. Wang.** 2001. Characterisation of p-nitrophenylglycerol-resistant Proteus mirabilis super-swarming mutants. Journal of medical microbiology **50:**1039-1048.

14. **Clemmer, K. M., and P. N. Rather.** 2008. The Lon protease regulates swarming motility and virulence gene expression in Proteus mirabilis. Journal of medical microbiology **57:**931-937.

15. **Wozniak C.E., L. C., and Hughes K.T.** 2009. T-POP array identifies EcnR and PefI-SrgD as novel regulators of flagellar gene expression J Bacteriol **191:**1498-1508.

16. **Wang Q., Z. Y., McClelland M., and Harshey R.M.** 2007. The RcsCDB signaling system and swarming motility in Salmonella enterica serovar Typhimurium: Dual regulation of flagellar and SPI-2 virulence genes J Bacteriol **189:**8447-8457.

17. **Wang, W. B., I. C. Chen, S. S. Jiang, H. R. Chen, C. Y. Hsu, P. R. Hsueh, W. B. Hsu, and S. J. Liaw.** 2008. Role of RppA in the regulation of polymyxin B susceptibility, swarming, and virulence factor expression in Proteus mirabilis. Infection and immunity **76:**2051-2062.

18. **Derzelle, S., E. Turlin, E. Duchaud, S. Pages, F. Kunst, A. Givaudan, and A. Danchin.** 2004. The PhoP-PhoQ two-component regulatory system of Photorhabdus luminescens is essential for virulence in insects. Journal of Bacteriology **186:**1270-1279.

19. **Alteri, C. J., J. R. Lindner, D. J. Reiss, S. N. Smith, and H. L. Mobley.** 2011. The broadly conserved regulator PhoP links pathogen virulence and membrane potential in Escherichia coli. Molecular microbiology **82:**145-163.

20. **Simms, A. N., and H. L. T. Mobley.** 2008. Multiple Genes Repress Motility in Uropathogenic Escherichia coli Constitutively Expressing Type 1 Fimbriae. Journal of Bacteriology **190:**3747-3756.

21. **Simm, R., U. Remminghorst, I. Ahmad, K. Zakikhany, and U. Romling.** 2009. A Role for the EAL-Like Protein STM1344 in Regulation of CsgD Expression and Motility in Salmonella enterica Serovar Typhimurium. Journal of Bacteriology **191:**3928-3937.

22. **Sharma V.K., B. S. M., and Bearson B.L.** 2010. Evaluation of the effects of sdiA, a luxR homologue, on adherence and motility of Escherichia coli O157:H7. Microbiology (Reading, England) **156:**1303-1312.

23. **Lee J., M. T., Hong S.H., and Wood T.K.** 2009. Reconfiguring the quorum-sensing regulator SdiA of Escherichia coli to control biofilm formation via indole and N-acylhomoserine lactones. Applied and Environmental Microbiology **75:**1703-1716.
